# Aberrant repair and fibrosis development in skeletal muscle

**DOI:** 10.1186/2044-5040-1-21

**Published:** 2011-05-04

**Authors:** Christopher J Mann, Eusebio Perdiguero, Yacine Kharraz, Susana Aguilar, Patrizia Pessina, Antonio L Serrano, Pura Muñoz-Cánoves

**Affiliations:** 1 Cell Biology Group, Department of Experimental and Health Sciences, Pompeu Fabra University (UPF), CIBER on Neurodegenerative diseases (CIBERNED), E-08003 Barcelona, Spain; 2 Institució Catalana de Recerca i Estudis Avançats (ICREA), Barcelona, Spain

## Abstract

The repair process of damaged tissue involves the coordinated activities of several cell types in response to local and systemic signals. Following acute tissue injury, infiltrating inflammatory cells and resident stem cells orchestrate their activities to restore tissue homeostasis. However, during chronic tissue damage, such as in muscular dystrophies, the inflammatory-cell infiltration and fibroblast activation persists, while the reparative capacity of stem cells (satellite cells) is attenuated. Abnormal dystrophic muscle repair and its end stage, fibrosis, represent the final common pathway of virtually all chronic neurodegenerative muscular diseases. As our understanding of the pathogenesis of muscle fibrosis has progressed, it has become evident that the muscle provides a useful model for the regulation of tissue repair by the local microenvironment, showing interplay among muscle-specific stem cells, inflammatory cells, fibroblasts and extracellular matrix components of the mammalian wound-healing response. This article reviews the emerging findings of the mechanisms that underlie normal versus aberrant muscle-tissue repair.

## Introduction

Pathophysiologic fibrosis, which is essentially an excessive accumulation of extracellular matrix (ECM) components, particularly collagen, is the end result of a cascade of events proceeding from tissue injury via inflammation, and resulting in permanent scar formation. Fibrosis can impair tissue function and cause chronic diseases in a large variety of vital organs and tissues, including bone marrow (BM). Despite the diverse range of tissues susceptible to fibrosis, all fibrotic reactions share common cellular and molecular mechanisms, such as cell and tissue degeneration, leukocyte infiltration, persistent inflammation of the tissue, and proliferation of cells with a fibroblast-like phenotype. The interplay and imbalance of different cell types sustains the production of numerous growth factors, proteolytic enzymes, angiogenic factors and fibrogenic cytokines, which together perturb the microenvironment of the damaged tissue, and stimulate the deposition of connective-tissue elements that progressively remodel, destroy and replace the normal tissue architecture. However, despite many common elements, there are also important differences between distinct tissue systems, and the identity of some cellular and soluble factors initiating and contributing to fibrogenic pathways are still unknown. Thus, improving our understanding of the mechanisms, cell types and factors involved in this process is crucial to develop treatment strategies for these diseases.

## The muscle tissue microenvironment controls normal repair versus fibrosis development

### Muscular dystrophies

In skeletal muscle, fibrosis is most often associated with the muscular dystrophies, a clinically and molecularly heterogeneous group of diseases. Phenotypically, these diseases are characterized by inflammation of the muscle tissue and skeletal-muscle wasting, which compromises patient mobility so that affected people become confined to a wheelchair. In the most severe cases, such as Duchenne muscular dystrophy (DMD, caused by the lack of the dystrophin protein), muscle loss and fibrosis also cause premature death through respiratory and cardiac failure [1]. In many dystrophies, including DMD, the mutation affects proteins that form a link between the cytoskeleton and the basal lamina, generally resulting in the disassembly of whole protein complexes. As a result, the sarcolemma becomes fragile, especially during intense contractile activity. In turn, there is focal or diffuse damage to the fiber and increased entry of calcium, although the underlying molecular mechanisms for these effects have not yet been elucidated in detail [2]. Several parallels can also be made between the muscular dystrophies and the idiopathic inflammatory myopathies (IIMs), which share common phenotypic features such as inflammation and muscle weakness, although the underlying causes are different.

**Figure 1:**
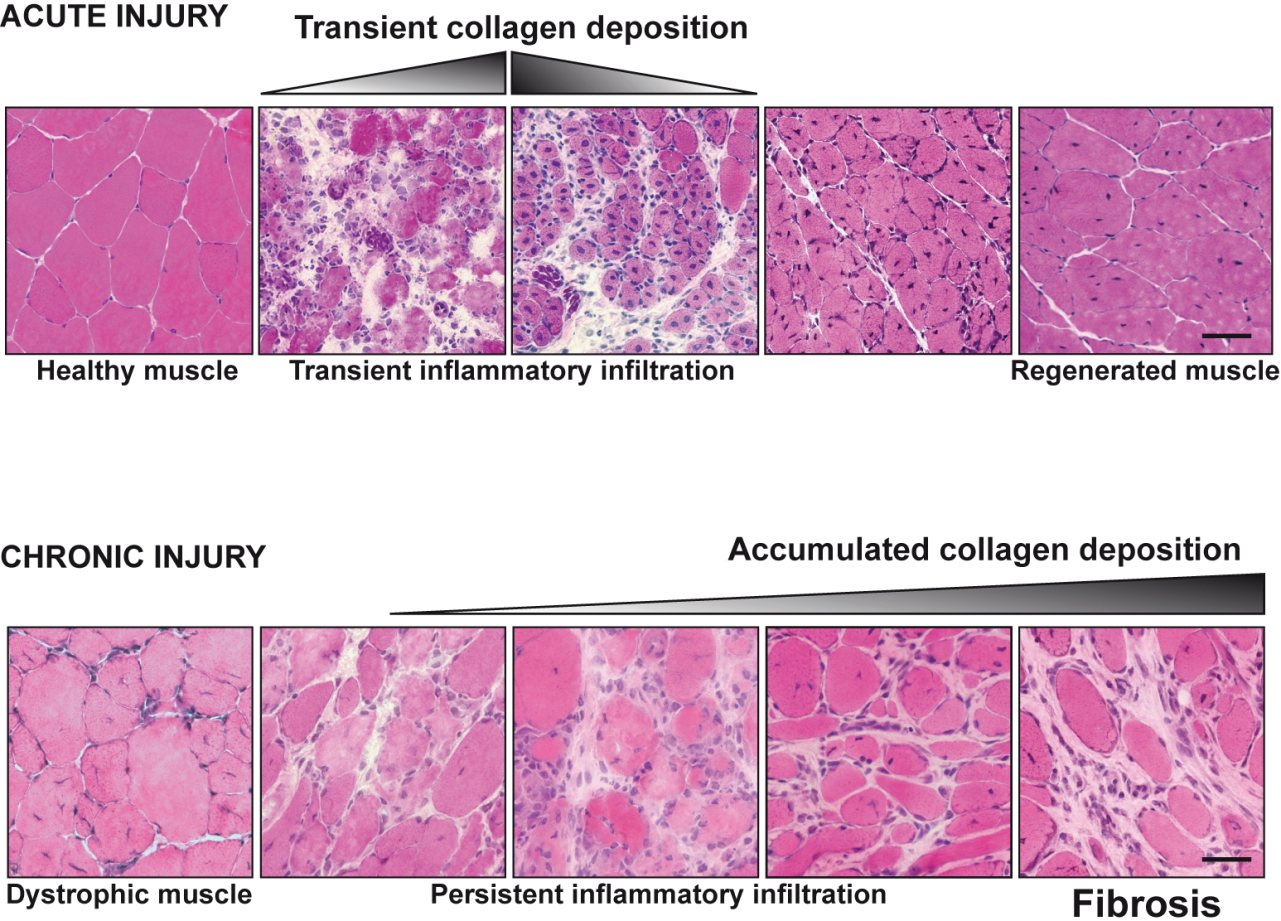
**Extracellular matrix (ECM) deposition in acute and chronic muscle regeneration**. Acute injury to healthy muscle produces rapid and controlled inflammation that removes dead and damaged myofibers, and promotes replacement of the injured muscle. However, in conditions of chronic injury, as occurs in the muscular dystrophies, chronic inflammatory events result in the excessive accumulation of ECM components, which inhibit myogenic repair and lead to muscle being replaced by fibrotic/scar tissue. (Top) Tibialis anterior muscles of mice were injected with cardiotoxin and samples were taken at different stages of the regeneration process. A representative sample showing the inflammatory phase, characterized by a transient increase in collagen deposition, and subsequently the resolving phase of healing, with progressive recovery of the normal tissue morphology (hematoxylin and eosin). (Bottom) Evolution of the morphological changes seen in the diaphragm of *mdx *dystrophic mice with disease progression, leading to heterogeneity in fiber size and increased collagen deposition between the altered myofibers. Bars = 50 μm.

In normal muscle repair after acute injury, such as in experimental animals and in humans after sports injuries, damaged or dead fibers are first removed by inflammatory cells, and they are then repaired or replaced by tissue-resident muscle stem cells known as satellite cells [3]. However, in chronic human diseases such as DMD and many other dystrophies, newly generated fibers are also prone to degeneration because they retain the underlying molecular defect, producing constant cycles of fiber degeneration associated with chronic inflammation (Figure 1) [4]. Until a few years ago, satellite cells were the only known post-natal regenerative cells with myogenic potential. In DMD, this satellite-cell population is either exhausted over time or it loses the capacity to mediate repair, and the muscle tissue is progressively replaced by adipose and fibrotic tissue. Fibrosis and loss of muscle tissue in dystrophies not only reduces motile and contractile functions, but also diminishes the amount of target tissue available for therapeutic intervention, or impairs the efficiency of these therapies [5]. Currently there is no effective therapy for DMD despite continuing efforts. The only relatively effective pharmacotherapy for DMD involves corticosteroid administration, which prolongs muscle strength and walking capacity in the early years, but eventually leads to undesirable secondary effects [6]. Furthermore, there is also no effective clinical treatment to combat or attenuate fibrosis in patients with DMD. For these reasons, recent studies using the *mdx *mouse model of DMD have focused more attention on the cellular and molecular mechanisms underlying fibrosis associated with dystrophin deficiency. Importantly, these studies have tested several pharmacological agents that target muscle fibrosis, and the results strongly suggest that combating the development of fibrosis could ameliorate DMD progression and increase the success of new cell- and gene-based therapies.

### Aging muscle

As well as the muscular dystrophies, aging is associated with loss of skeletal-muscle mass and function with concomitant fibrosis and ECM deposition. Age-associated muscle loss (sarcopenia) causes and/or exacerbates age-related health problems. Therefore, understanding the processes involved is important not only for unraveling the mechanisms of fibrosis, but also for improving quality of life and healthcare for the older person. Sarcopenia seems to occur by mechanisms that partly are unique to it, and partly are common to other forms of atrophy. Some of these may involve changes in soluble effectors, such as altered hormone status, inflammatory factors, and altered caloric and protein intake, perhaps triggered by modifications or decline in the central and peripheral nervous systems. The net consequence of these alterations firstly involves progressive atrophy and loss of individual muscle fibers, associated with concomitant loss of motor units [7]. In addition, there is infiltration of fat and other non-contractile material, which causes a reduction in muscle 'quality' [8]. At the ultrastructural level, aging has also been associated with myofibril disarrangements in a dystrophic animal model, and drops in force without alterations in motor protein function as measured by *in vitro *motility assays [9]. Additional factors associated with DMD and age-associated fibrosis are discussed in further detail below.

### Normal skeletal-muscle repair

**Figure 2:**
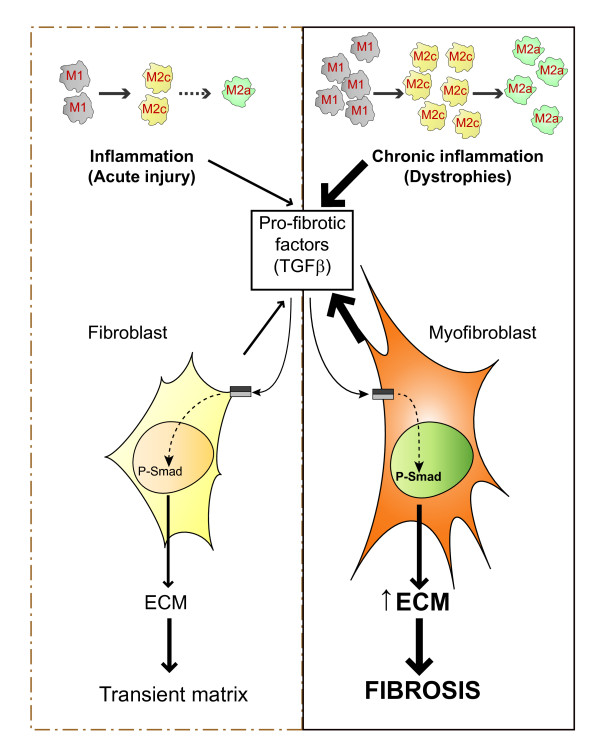
**Chronic inflammation leads to fibrosis in skeletal-muscle repair**. Resident and extravasating peripheral macrophages play an important role in the early stages of muscle repair after acute injury, with pro-inflammatory (M1) macrophages first acting to clear the damage, and anti-inflammatory (M2c) macrophages and alternatively activated macrophages (M2a), implicated in the subsequent resumption of inflammation, extracellular matrix (ECM) deposition and tissue repair. M2c and M2a macrophages release anti-inflammatory cytokines and pro-fibrotic molecules such as transforming growth factor (TGF)-β, which in turn activate fibroblasts in a regulated manner to produce ECM components and ECM-remodeling factors, including autocrine production of TGFβ, collagen, fibronectin, serine proteases (such as uPA/plasmin), and metalloproteinases (MMPs) and their inhibitors (TIMPs). However, during chronic tissue damage, as in muscular dystrophies, the increased and persistent presence of macrophages modify the intensity, duration and interactions of these released factors, leading to excessive ECM accumulation and replacement of muscle with fibrotic tissue.

**Figure 3:**
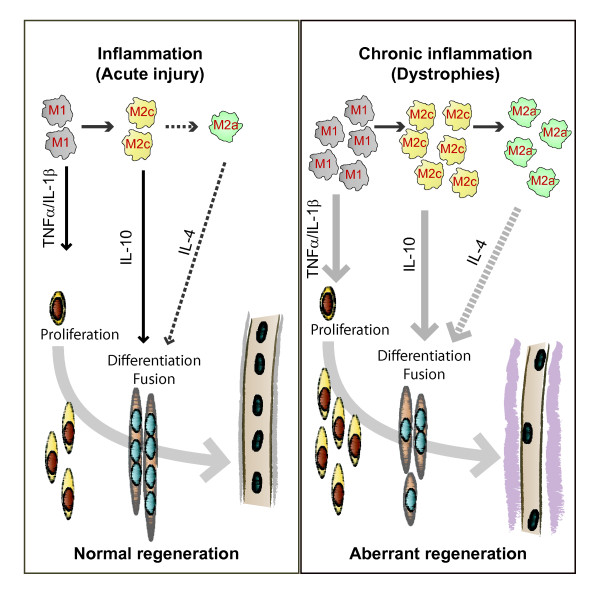
**Inflammatory control of skeletal-muscle regeneration**. Replacement of damaged muscle fibers is dependent on satellite cells, resident stem cells that are normally quiescent, and are located under the basal lamina of muscle fibers. Tissue damage leads to their activation, proliferation, differentiation and fusion to form new myofibers. However, their capacity to mediate repair is modified by the extent and type of injury, and consequently by their interaction with various cellular and soluble mediators, most importantly with infiltrating macrophages. The proposed paracrine interaction between macrophages and satellite cells is as follows. During the timely, regulated process of regeneration after acute injury (left), pro-inflammatory cytokines released from M1-macrophages may promote satellite-cell proliferation, whereas cytokines released by anti-inflammatory (M2c) and alternatively activated (M2a) macrophages, respectively, may favor their differentiation and fusion. In particular, interleukin (IL)-4 was shown to regulate fusion of myoblasts *in vitro *and *in vivo *[129]. It could be expected that, during chronic damage (right), such as in muscular dystrophies, the increased and persistent presence of the distinct macrophage cell types could modify the relative levels and kinetics of these cytokines, resulting in altered satellite-cell functions and aberrant regeneration, with progressive development of fibrosis and fat accumulation, ultimately leading to non-functional muscle tissue.

In nature, survival of an organism can often depend on the ability to rapidly repair damage to muscle from mechanical trauma, exposure to toxins or infections. This rapid resolution of tissue injury requires a sequential and well-orchestrated series of events. Perturbation of any of these stages can result in unsuccessful muscle regeneration, typically characterized by persistent degeneration of myofibers, inflammation and fibrosis [10-12]. The key events leading to normal and defective/fibrotic muscle repair are shown in Figure 2 and 3, and detailed below.

Immediately after skeletal-muscle injury, cytokines and growth factors are released from both the injured blood vessels and from infiltrating inflammatory cells [13,14]. These factors stimulate the migration of the inflammatory cells to and at the site of injury, and mediate proliferation and cell survival. Invading inflammatory cells are also responsible for phagocytosing any cell debris. The specific influence of many damage signals, growth factors and inflammatory molecules on satellite cells remains unclear [14], but the next crucial stage of repair is the formation of new muscle fibers by these cells. This process begins with their activation, because satellite cells normally lie in a quiescent state beneath the basal lamina of muscle fibers, followed by their extensive proliferation. Some cells undergo self-renewal to replenish the satellite-cell pool, but most become committed and subsequently differentiate. These later myoblasts fuse either to themselves or to the damaged myofibers to replace the lost muscle.

In addition to inflammatory and satellite cells, efficient muscle repair also requires the migration and proliferation of fibroblasts, in order to produce new temporary ECM components, such as collagen types I and III, fibronectin, elastin, proteoglycans, and laminin. These elements serve to stabilize the tissue, and they act as a scaffold for the new fibers. Moreover, the satellite cells also utilize the basement membranes of pre-existing necrotic fibers to ensure the myofiber maintains a similar position. Basement membranes and temporary ECM components are also crucial for guiding the formation of neuromuscular junctions (NMJs) [15]. The formation and degradation of the ECM is mediated by the expression of proteases and their specific inhibitors during tissue repair. ECM degradation also leads to the generation of protein fragments that mediate important biological activities required to facilitate normal tissue repair [16]. Finally, in addition to ECM remodeling, angiogenesis facilitates the development of a new vascular network at the site of injury, while newly formed muscle fibers undergo growth and maturation.

### Inflammation in efficient muscle repair and fibrosis

The first event after muscle damage is the invasion of the injury site by inflammatory cells. There is now a wealth of evidence to suggest that the nature, duration and intensity of the inflammatory response after muscle damage and regeneration can crucially influence the outcome of muscle repair, or alternatively, fibrosis [12,14,17,18]. For example, interfering with the transient inflammatory response after acute injury may negatively affect the phagocytosis of dead and damaged fibers, thereby impeding the formation of new tissue. By contrast, modulating the chronically high levels of inflammation in dystrophic muscle can be beneficial in reducing both muscle degeneration and fibrosis, while simultaneously promoting regeneration [19].

These results highlight two key notions: firstly, that some form of inflammatory response is necessary to repair damaged tissues effectively; and secondly, that chronic inflammatory responses drive unrestrained wound healing and fibrosis.

### The inflammatory response: role of different macrophage populations

The earliest phases of tissue repair are generally characterized by local activation of the innate immune system, even though the original immunogenic stimuli are not always known (see below) [20]. Macrophages have a prominent role in the innate immune response to infection and/or tissue injury, because of their ability to phagocytose particles such as bacteria or cellular debris, and to secrete pro-inflammatory cytokines [13]. Recent studies have shown that resident macrophages in the muscle epimysium/perimysium orchestrate the innate immune response to injury, which is linked to adaptive immunity through inflammatory dendritic cells [21]. In addition to tissue-resident macrophages, invasion of the site of damage involves both polymorphonuclear leukocytes (for example, neutrophils) and blood-derived monocytes, which also differentiate into macrophages [17]. In some tissues, other inflammatory-cell types such as mast cells and T cells also play a key role in repair and fibrogenesis, although to date there are only limited studies into the role of these cells in muscle repair and DMD [22].

The principal inflammatory cells present in injured muscle are monocytes and macrophages [17]. In regenerating and dystrophic muscle, these serve to clear myofiber debris and in part, they modulate regeneration by secreting cytokines. An important development in our understanding of muscle repair and fibrosis was the demonstration that a heterogeneous population of macrophages exists in regenerating muscle after injury, exhibiting opposing activities (either pro-inflammatory or anti-inflammatory) and different kinetics [23]. A nomenclature for polarized macrophages has been proposed [24,25] and they are now referred to as classically and alternatively activated macrophages, or M1 and M2 macrophages, respectively (Figure 2, Figure 3).

Classically activated (M1) or pro-inflammatory macrophages, arise from exposure to the T-helper (Th)1 cytokines interferon-(IFN)γ and tumor necrosis factor (TNF)-α, in addition to lipopolysaccharide (LPS) or endotoxin [24-26]. M1 macrophages play a key role in acute inflammatory processes, and they are therefore considered to be the prototypic macrophage. They are found during the early stages after muscle damage in association with recruited monocytes, and they participate in the processing and presentation of antigens, and in the phagocytic removal of necrotic material. M1 macrophages also produce high levels of pro-inflammatory cytokines, such as TNFα and interleukin (IL)-1β and IL-12. In addition, they can be induced to express nitric oxide synthase (iNOS; also known as NOS2), which is required to efficiently metabolize L-arginine to generate the large amounts of NO involved in killing intracellular pathogens.

**Figure 4:**
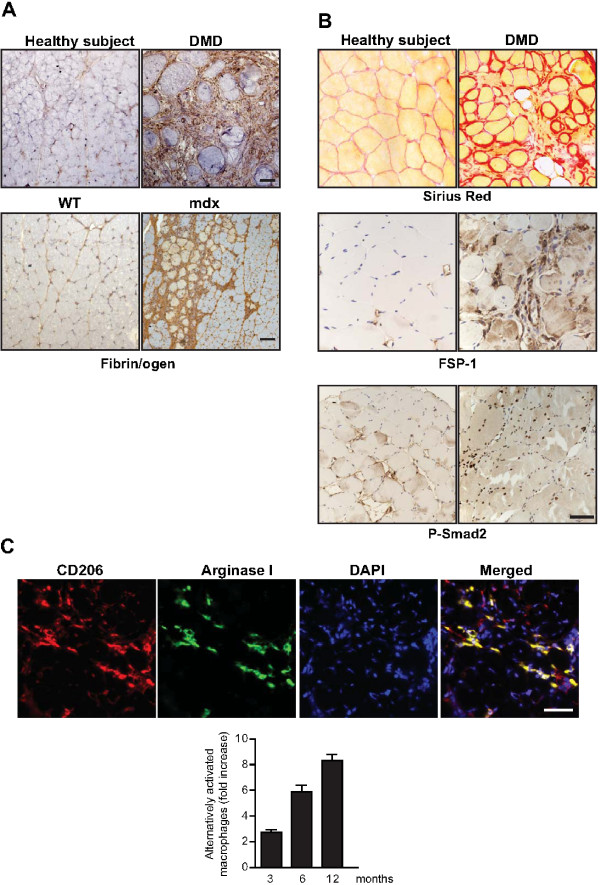
**Inflammatory and fibrotic traits in dystrophic muscle of patients with DMD and *mdx *mice**. **(A) **Fibrin(ogen) accumulates in muscles of patients with DMD and in aged *mdx *mice. Immunohistochemistry for fibrin(ogen) (brown) in muscle biopsies of (top) patients with DMD and healthy subjects and in (bottom) wild-type (WT) and *mdx *diaphragms. **(B) **Increased fibrosis, fibroblast number and TGFβ signaling in dystrophic muscles. Staining for collagen deposition (Sirius red) and immunohistochemistry for fibroblast-specific protein (FSP)-1 and P-Smad2 was performed on muscle biopsies taken from patients with DMD and healthy subjects. **(C) **Presence of alternatively activated macrophages in diaphragm muscle of *mdx *mice. Cells double-positive for CD206 (red) and Arginase I (green) in *mdx *diaphragm are shown by immunofluorescence with specific antibodies. The relative increase in the number of these cells in the *mdx *diaphragm over time is shown. Bars = 50 μm.

The population of M2 macrophages is more complex than that of the M1 macrophages, and it is currently divided into distinct subtypes, reflecting different functional specializations. M2a macrophages or strictly speaking, alternatively activated macrophages, are activated by the Th2 cytokines IL-4 and IL-13, and are most commonly associated with tissue repair, wound healing and fibrosis. M2c macrophages are considered to be anti-inflammatory, because they play a key role in deactivating the M1 phenotype and they promote the proliferation of non-myeloid cells. M2c macrophages release anti-inflammatory cytokines, and they are primed by IL-10. Thus, classically activated M1 macrophages are usually found in the early stages after muscle injury, closely followed by M2c macrophages [23]. M2a macrophages are abundant in advanced stages of the tissue-repair process [12], and have been found in fibrotic muscle of *mdx *mice [27,28] (Figure 4C).

### From muscle injury to the chronic inflammatory response and pathological muscle fibrosis

Alterations in the intensity or duration of macrophage responses can have profound effects on muscle regeneration and fibrosis. One example of this was the deletion of IL-10 in *mdx *mice, which increased muscle damage and reduced muscle strength, due to an imbalance between M1 and M2 macrophages [29]. Similarly, persistence of M1 macrophages has been proposed to have pathological consequences in chronic inflammatory myopathies (see below). In terms of tissue fibrosis, M2a macrophages are generally considered the most important [12]. They express specific cell surface markers such as the mannose receptor CD206 and the type II IL-1 decoy receptor, in addition to releasing a range of regulatory cytokines such as IL-10 and the soluble IL-1 receptor antagonist (IL-1Ra) as well as many pro-fibrotic molecules such as transforming growth factor (TGF)-β, fibronectin, proline, several types of tissue inhibitor of matrix metalloproteinases (TIMP) and chemokine (C-C motif) ligand (CCL)17. CCL17 in particular has been shown to enhance fibrosis in several mouse models of pulmonary disease by binding to CC chemokine receptor (CCR)4 [30]. An additional reason for the ability of M2 macrophages to neutralize the M1 pro-inflammatory response is their high level of expression of arginase (ARG)1, which directly competes with M1-associated inducible nitric oxide synthase (iNOS) for L-arginine [31] (see below and Figure 4C).

Several groups have used different *in vitro *and *in viv*o animal models in attempts to unravel the role of macrophages in myogenesis, muscle repair, fibrosis, and the development and treatment of DMD. *In vitro*, pro-inflammatory macrophages have a positive influence on myoblast proliferation while repressing myoblast differentiation, whereas anti-inflammatory macrophages stimulate both myoblast differentiation and fusion. Importantly, *in vivo *depletion of blood monocytes, from which M1 and M2 macrophages probably arise, was shown to have negative effects on the muscle-repair process after injury [23]. Conversely, BM transplantation experiments have revealed an important role for the CC chemokine receptor 2/monocyte chemotactic protein-1 (CCR2/MCP-1) ligand, and for the proteolytic activity of urokinase plasminogen activator (uPA)/plasmin in skeletal-muscle repair by regulating the recruitment of BM-derived macrophages into injured muscle [32-37].

Several studies of macrophage depletion or impaired macrophage recruitment have revealed crucial functions of macrophages in the regulation of fibrogenesis in dystrophic muscle, and the potential for therapeutic intervention [19,35,37,38]. Similar benefits on the dystrophic phenotype in *mdx *mice were reported after using a variety of anti-inflammatory agents acting on cytokines such as TNFα and on their cellular receptors, or on other major pro-inflammatory mediators such as nuclear factor (NF)-κB [39-41]. Additionally, the presence of alternatively activated M2a macrophages was shown to increase progressively with age in the diaphragms of fibrotic *mdx *mice [27] (Figure 4C). Similarly, fibrinogen depletion in *mdx *mice diminished fibrosis, concomitant with a significant decrease in the number of M2a macrophages in the diaphragm. In patients with DMD, fibrosis has also been associated with increased numbers of alternatively activated macrophages [42]. Overall, these studies show that appropriate modulation of macrophage activity might ameliorate the progression of dystrophy.

A potentially important link between arginine metabolism by M2a macrophages and abnormal repair or fibrosis development has recently been shown by several groups. In one study, deletion of two c-AMP response element (CREB)-binding sites from the C/EBP-β promoter specifically impaired M2 but not M1 gene expression, interfering with the later stages of injury-induced muscle regeneration. Mutant mice were able to remove necrotic tissue from injured muscle, but they exhibited severe defects in myofiber regeneration [43]. Mutation of the C/EBP-β promoter also reduced ARG1 expression in macrophages, which was hypothesized to reroute arginine metabolism away from arginase-mediated polyamine synthesis toward iNOS-mediated NO production, which was previously shown to promote the degradation of the key myogenic transcription factor MyoD [44]. Indeed, similar shifts in macrophage polarization and macrophage competition for arginine metabolism were seen to influence the severity of the muscle pathology in *mdx *dystrophic mice [28]. In another study [14], Th2 cytokines increased the expression and activity of arginase by M2 macrophages in *mdx *mice, with intriguing differences in the effects of arginase-2 deletion in different muscles. Indeed, although fibrosis is reduced in quadriceps and diaphragm of *mdx *mice lacking arginase-2, it seems that it is not the case for the soleus, the cardiac muscle and the longissimus dorsi. More importantly from the clinical perspective, long-term dietary supplementation with arginine increased skeletal and cardiac muscle fibrosis in dystrophic mice, in contrast to the reported benefits from short-term supplementation, thus suggesting caution is needed regarding dietary arginine supplementation for patients with DMD [45]. Taken together, these studies indicate the crucial roles of macrophage polarization in both muscle repair and fibrogenesis, particularly in dystrophic muscle.

### Aberrant repair and fibrosis in IIMs

In addition to the muscular dystrophies, there is another group of chronic muscle disorders, collectively known as myositis or the IIMs [46]. Phenotypically, IIMs are characterized by muscle weakness, poor endurance, and ongoing regeneration of the muscle tissue. Within the muscle, the presence of inflammatory infiltrates composed largely of macrophages, T cells and dendritic cells correlates with immune-mediated loss of muscle fibers and an inability to resolve the regeneration process effectively. Although not fully characterized, an important feature of these diseases is the persistence of pro-inflammatory M1 macrophages and associated cytokines such as IL-1, IL-15 and TNFα in the tissue, and an apparent inability to switch to anti-inflammatory M2 macrophages. Hence, the most effective treatments to date have proven to be glucocorticoids and other nonsteroidal anti-inflammatory agents (NSAIDs) such as ibuprofen and aspirin. However, one problem with these drugs has been their ability to promote fiber atrophy [47], in addition to their negative effect on prostaglandin (PG) synthesis via interference with cyclooxygenase (COX) gene functions. PGs have been shown to play various roles in many stages of myogenesis, and are secreted from regenerating muscle [48,49]. Interestingly, macrophage-derived TGFβ1 has been shown to induce PGE_2 _expression from myoblasts via a COX-2-dependent mechanism, which in turn diminishes TGFβ1 expression, forming an important feedback loop that can control inflammation and fibrosis development [49]. One other limitation of the drugs used to treat IIMs is that they do not produce complete recovery, nor do they address the underlying defects, which in most cases are multifactorial and not fully defined [50]. Nonetheless, the IIMs represent an important group of diseases to help us understand the complicated role of pro-inflammatory cells and cytokines in orchestrating normal versus aberrant muscle repair.

### The role of other immune-cell types in muscle repair and fibrosis

Macrophages are not the only cells in the immune system known to play a role in muscle repair and dystrophy. Tissue repair and fibrosis is also tightly regulated by the Th cell response. Like macrophages, T lymphocytes can differentiate into different functional types, which are named Th1 and Th2 cells, and which orchestrate the host response by generating distinct cytokine profiles [26]. CD4+ Th1 cells produce cytokines that promote cell-mediated immunity, including IFN-γ, TNFα, IL-12 and IL-2, all of which have been found to be anti-fibrotic cytokines. By contrast, CD4+ Th2 cells promote humoral immunity, and produce the pro-fibrotic cytokines IL-4, IL-5, IL-6 and IL-13. Th1 cytokines inhibit the development of Th2 cells, and conversely, Th2 cytokines inhibit the development of Th1 cells. Clearly, alterations or imbalances in these pathways have the potential to skew repair towards anti- or pro-fibrotic pathways, as witnessed by the importance of Th2 cytokines in the development of liver fibrogenesis [12]. T-cell-produced cytokines also regulate muscle degeneration and repair. For example, loss of uPA proteolytic activity in transgenic knockout mice reduces macrophage and T-lymphocyte infiltration of injured muscle, in association with more persistent myofiber degeneration [33]. Moreover, *scid/mdx *mice, which are deficient in functional T and B lymphocytes, develop much less diaphragm fibrosis at 1 year of age, concomitant with a decrease in activated TGFβ in skeletal muscle, compared with normal *mdx *mice [51]. In *nu/nu/mdx *mice, (immunodeficient nude mice in the *mdx *background) the lack of functional T cells alone was associated with less diaphragm fibrosis at 3 months, supporting the pathogenic role for T cells in *mdx *muscle, and revealing this lymphocyte subclass to be an important source of TGFβ1 [52].

A specific subpopulation of T cells expressing the Vβ8.1/8.2 T-cell receptor (TCR) was recently identified and shown to be enriched in *mdx *muscle. These T cells produce high levels of osteopontin, a cytokine that promotes immune-cell migration and survival [53], and osteopontin levels are increased in patients with DMD and in *mdx *mice after disease onset. Importantly, loss of osteopontin in *mdx *double-mutant mice diminishes the infiltration of natural killer T-cell (NKT)-like cells, which express both T and NK cell markers and neutrophils, in addition to reducing the levels of TGFβ. These results correlated well with improvements in muscle strength and reduced diaphragmatic and cardiac fibrosis [53]. Not all studies have produced such definitive results, and the implication of lymphocytes and their subtypes in muscle repair and fibrosis clearly requires further study. For example, thymectomy at 1 month of age induces near-complete post-natal depletion of circulating T cells in *mdx *mice and. when followed by anti-CD4 and/or anti-CD8 antibody treatment, failed to improve diaphragm fibrosis at 6 months of age in *mdx *mice [51,54,55]. In another study, M2 macrophages were shown to influence CD4+ Th cells, because ARG1-expressing macrophages suppressed Th2 cytokine-driven inflammation and fibrosis in the liver induced by *Schistosoma mansoni *infection [26]. Because these data demonstrate the complexity of the mechanisms regulating inflammation and fibrosis development, further studies are clearly necessary to determine whether distinct types of Th responses and macrophage subtypes operate in dystrophic muscle. and how they mediate their interactions.

### Fibroblasts, the collagen-producing cells in skeletal muscle

When tissue is damaged, fibroblasts migrate into the wound and begin to produce and remodel the ECM in response to pro-fibrotic cytokines such as TGFβ. Stromal fibroblasts produce cytokines, growth factors and proteases that trigger and uphold acute and chronic inflammatory/pro-fibrotic conditions. Indeed, although the fibroblast is necessary and fundamental to tissue homeostasis and normal wound repair, it is also a crucial intermediate in chronic fibrotic diseases, in which persistent inflammation is widely accepted to provoke dysregulated fibroblast activity. Notably, one limitation that has hindered studies of fibrotic disease has been the lack of good genetic markers to label fibroblasts. It is well established that in non-muscle systems. activated fibroblasts may be identified by their increased proliferation, migratory ability, enhanced contractility. and increased expression of vimentin and, in particular, α-smooth-muscle actin (αSMA), a contractile protein of stress fibers. These fibers are connected to the ECM through specialized structures called 'mature' and 'super-mature' focal adhesions, and through intercellular gap and adherent junctions. As a result, when αSMA stress fibers contract, they exert mechanical tension on the ECM, which in turn provides a mechanically resistant support, hence the name 'myofibroblast'. These cells are associated with tissue repair and fibrosis in many tissues and organs, including muscle, skin, liver, lung, bone and cartilage [56]. However, despite their relevance in these diseases, it remains unclear whether myofibroblasts really do exist in fibrotic skeletal muscle, or whether they are instead mature fibroblasts actively producing ECM components. One reason for this controversy is that classic markers such as vimentin or αSMA are also expressed by myoblasts, albeit at lower levels than fibroblasts. However, a recent study has identified the transcription factor Tcf4 as a potentially important marker of fibroblasts in muscle, although follow-up studies are still needed to validate its utility [57]. Nonetheless, to remain consistent with the literature, we use the term 'myofibroblast' here in the muscle context.

There are many possible origins of myofibroblasts. Resident fibroblasts that differentiate in response to specific effectors are considered to be the main myofibroblast progenitor in most tissues. Alternatively, myofibroblasts may arrive by the influx of circulating BM-derived cells expressing CD34, CD45 and collagen I (cells known as fibrocytes), which undergo further reprogramming in the damaged area. Finally, it has been shown in organs such as lung, kidney and liver that there is a significant number of myofibroblasts derived from parenchymal epithelial cells through the mechanism of epithelial to mesenchymal transition (EMT). Thus, transdifferentiation of distinct cell types into myofibroblasts can potentially account for persistent ECM deposition in chronically damaged tissues. Understanding the origin of myofibroblasts is thus of great importance to develop new approaches to combat the fibrotic process seen in diverse diseases.

Although fibroblasts are the major collagen-producing cells, myofiber-associated satellite cells and C2C12 myoblasts have also been shown to express significant levels of interstitial collagens I and III, which diminish during the process of differentiation [58]. Whereas collagen I can markedly suppress differentiation of C2C12 cells, collagen III expression is retained in aged *mdx *myogenic cells. This suggests that conversion of myoblasts into myofibroblasts with increasing age may occur via positive feedback [58]. Collagen modifications, such as non-enzymatically regulated crosslinking to produce advanced glycation end (AGE) products, also increase the stiffness of muscle connective tissue, thereby contributing to impaired muscle function in the older person [59]. Several recent studies in other models have also investigated the induction of fibroblastic phenotypes in myogenic cells. In one case, TGFβ was able to induce Smad-dependent upregulation of sphingosine kinase SK)1 in C2C12 myoblasts, whereas pharmacological or small interfering (si)RNA-mediated inhibition of SK1 prevented TGFβ from inducing fibrotic markers. Rho/Rho kinase signaling also appeared to be implicated in the TGFβ-mediated transition of myoblasts into myofibroblasts downstream of SK1 activation [60]. Similarly, downregulation of Notch2 expression has also been linked to non-muscle fibrotic tissue and TGFβ-dependent induction of myofibroblast markers in C2C12 myoblasts. Overexpression of active Notch2 in C2C12 cells prevents TGFβ from inducing the expression of αSMA and collagen I, whereas more surprisingly, transient knockdown of Notch2 by siRNA in cultured myoblasts results in the differentiation of C2C12 myoblasts into myofibroblastic cells that express fibrotic markers such as αSMA and collagen I, even in the absence of TGFβ. Finally, Notch2 can inhibit the differentiation of myoblasts into myofibroblasts by directly counter-regulating Notch3 and limiting its expression [61].

### Fibrogenesis in aging muscle

The Notch pathway has also been strongly implicated in aging-associated fibrosis. For example, analysis of the microniche of aged murine muscle stem cells found high levels of TGFβ and its activated effector Smad3 in both differentiated muscle fibers and satellite cells, which was reciprocal to the levels of active Notch, which is more abundant in the young microniche [62]. Increased levels of activated Smad3 levels in aged muscles attenuate their regenerative capacity by binding to the promoters and stimulating the expression of several cyclin-dependent kinase (CDK) inhibitors (for example,, p15, p16, p21 and p27), negative regulators of cell-cycle progression. Importantly, this imbalance of TGFβ/pSmad3-Notch could be restored by forced activation of Notch. Similar scenarios of reduced Notch activation and increased TGFβ/pSmad3 signaling have been reported recently in aged human muscles [63].

In additional studies of aged muscle, the fate of muscle stem-cell progeny was reported to be controlled by an interaction between the Wnt and Notch pathways in which glycogen synthase kinase (GSK)3β plays an important role [64]. The mammalian ortholog of the *Drosophila *transcriptional coactivator Legless, BCL9/9-2, was also shown in this study to be necessary for activation of the canonical Wnt pathway in adult myogenic progenitors, and for their Wnt-mediated commitment to differentiation and effective muscle regeneration [64]. However, whether GSK3β and/or BCL9 mediate Wnt-induced cell-fate changes from myogenic to fibrogenic lineages in resting satellite cells awaits further validation. An earlier study by the same group had already highlighted the role of the canonical Wnt pathway in age-associated fibrosis, linking increased collagen deposition in aged regenerating muscles to a greater percentage of fibrogenic cells arising from the conversion of myogenic into non-myogenic cells [65]. This fibrogenic conversion could be abrogated experimentally by treating mice with Wnt inhibitors. Wnt3A stimulation negatively modulated cell proliferation in young regenerating muscles, augmenting fibrosis. Thus, aging was associated with alterations in the systemic environment, and because these effects were reversible, this work provides the strategic basis for interventions aimed at improving tissue repair and at reducing fibrosis in pathological conditions.

### Fibrogenesis versus adipogenesis in muscle repair

**Figure 5:**
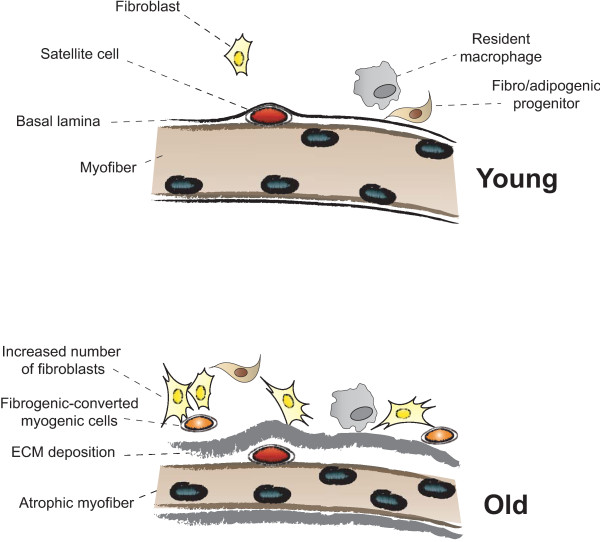
**Myofiber growth and extracellular matrix (ECM) accumulation after damage differ in young and aged muscles**. (Top) In young muscles in response to injury, the satellite cells under the basal lamina can be activated by environmental cues released by the neighboring cells (a local milieu composed of fibroblasts, interstitial cells, resident macrophages, fibro/adipogenic progenitors (FAPs) and microvasculature-associated cells) to eventually form new fibers almost indistinguishable to the pre-existing ones. (Bottom) In aged muscles, the repair process will result in reduced size of newly formed myofibers and thickening of the basal lamina by enhanced deposition of ECM components, which could potentially be ascribed to the increased presence and activity of resident fibroblasts (and/or FAPs), and to decreased satellite-cell myogenic potential. Conversion of myogenic into fibrogenic cells could also contribute to ECM accumulation. These new microenvironment conditions within the satellite-cell niche will impede efficient satellite-cell functions and muscle repair.

It is known that when regeneration fails, the fibrotic scar is infiltrated with adipocytes (fatty degeneration) in addition to fibroblasts [66]. However, the cellular origin of fatty infiltration remains controversial. Two independent contributions to this debate have recently been published, which identified a novel type of resident muscle cell that responds to damage in skeletal muscle, termed the fibro/adipogenic progenitor (FAP) [67,68]. These cells express the mesenchymal marker platelet-derived growth-factor receptor (PDGFR)-α and are not myogenic either *in vitro *or *in vivo*, according to their distinct embryonic origin. FAPs are thought to be a source of pro-differentiation signals for myoblasts during the process of muscle regeneration, and more importantly, they show a strong tendency to generate myofibroblasts and adipose cells [67,68]. Thus, although FAPs display limited myogenic capacity both *in vitro *and *in vivo*, the FAP population will persist in the tissue if the regenerative process fails, and they will potentially differentiate into adipocytes. This supports the view that signals from the local environment help control FAP fate, and may play a significant role in the successful regeneration of healthy muscle (Figure 5). Whether FAP conversion also occurs during aging, and whether systemic factors such as Wnt ligands also contribute to this process, remain to be determined. Interestingly, there is the suggestion that the type of injury in acute models may drive the repair response towards more fibrotic or more adipogenic repair, for example after glycerol injury and acute ischemia, respectively [68,69], although the mechanisms have not been extensively investigated.

### The role of TGFβ in muscle repair and fibrosis

Most pro-fibrogenic polypeptides are produced by infiltrating immune, inflammatory, mesenchymal and tissue-specific cells, thereby facilitating paracrine pro-fibrogenic effects that perpetuate inflammation-driven fibrosis (see below) (Figure 2, Figure 3) [12]. One of the most potent pro-fibrogenic cytokines *in vivo *is TGFβ. Three TGFβ isoforms have been described to date (TGFβ1, TGFβ2 and TGFβ3), all of which are initially generated as latent precursors [70]. When active TGFβ is liberated, it binds to a heterodimeric receptor complex consisting of one TGFβ type I receptor molecule, termed activin-linked kinase (ALK)5, and one TGFβ type II receptor. In the canonical TGFβ pathway in normal fibroblasts, ligand binding leads ALK5 to phosphorylate Smad2 and 3, which in turn bind to Smad4 to form a complex that is translocated to the nucleus, which activates transcription [71]. However, TGFβ has also been shown to signal via several additional pathways, including p38 mitogen-activated protein kinase (MAPK), which requires the heparin sulfate-containing proteoglycan (HSPG) betaglycan; the Ras/MAPK kinase (MEK)/extracellular signal-regulated kinase (ERK) pathway, which requires the HSPG syndecan 4; the c-abl pathway; and Jun kinase (JNK), which requires focal adhesion kinase (FAK) and TGFβ-activated kinase (TAK)1 [72]. These signaling pathways seem to eventually modify gene expression in a promoter-selective fashion. For example, FAK, JNK and TAK1 are required for myofibroblast differentiation and α-SMA expression, whereas ERK is required for collagen type I expression. However, p38 MAPK does not seem to be involved in the fibrogenic activity of TGFβ. Thus, it is likely that additional signaling pathways are abnormally activated in muscle fibroblasts in a manner independent of the canonical TGFβ pathway.

TGFβ is expressed in normal muscle after injury and in the dystrophic muscle of patients with DMD and *mdx *mice [70], where it has the potential to induce fibrosis around myofibers, probably by stimulating fibroblasts to produce ECM proteins such as collagen and fibronectin. Equally important is the ability of TGFβ to decrease the production of enzymes that degrade the ECM, such as collagenase, while increasing production of proteins that inhibit ECM-degrading enzymes such as TIMPs and plasminogen activator inhibitor (PAI)-1 (see below). Direct injection of recombinant TGFβ into skeletal muscle *in vivo *stimulates TGFβ expression in myogenic cells in an autocrine fashion and induces connective-tissue formation in the area of the injection [73,74]. Moreover, myoblasts transfected with vectors expressing TGFβ can differentiate into myofibroblastic cells after intramuscular transplantation, a process inhibited by decorin, a small leucine-rich proteoglycan that can bind to and inhibit TGFβ (see also below) [75,76]. Finally, latent TGFβ-binding protein (LTBP)4 regulates the release and bioavailability of TGFβ from the ECM, where it has been shown to modulate fibrosis in the context of muscular dystrophy in mice [77] Taken together, these studies highlight the crucial role of TGFβ in the initiation of fibrotic processes, and in the induction of differentiation of myogenic cells into myofibroblastic cells in injured muscle.

TGFβ also seems to play an important role in aging-associated fibrosis and muscle impairment. Increased TGFβ levels have been reported during aging, possibly activating SK1 signaling to trigger fibrosis [60]. Microarray analyses of myogenic cells from aged animals also revealed major alterations in the expression of many genes dependent upon the activation of TGFβ signaling [78]. In particular, PAI-1, fibronectin and connective-tissue growth factor (CTGF), which are all known to be directly upregulated by TGFβ, were upregulated in aged myogenic cells, together with increased basal levels of phosphorylated Smad2/3. Collectively, these data suggest that TGFβ signaling is constitutively active in aged myogenic progenitors, which may explain the increase in fibrosis seen in aged muscles.

Antagonism of TGFβ signaling by a number of therapeutic agents has been shown to inhibit fibrosis and improve muscle regeneration in several experimental and animal models. However, no agents have yet been convincingly shown to reduce fibrosis once formed. For example, direct immunomodulation of TGFβ inhibited connective-tissue accumulation and the progression of fibrosis in the diaphragm of *mdx *mice, although inflammation increased [79]. Reduced tissue fibrosis after injury was also detected in mice treated with both decorin- and angiotensin receptor antagonists, with decorin also preventing TGFβ-induced differentiation of myogenic cells into fibrotic cells [80]. Likewise, extended treatment of *mdx *mice with losartan significantly reduces diaphragmatic fibrosis with no apparent adverse effects [81]. Similar results were reported in animals treated with suramin, which acts by competing with TGFβ for receptor binding [82]. Finally, the administration of halofuginone to aged *mdx *mice improved cardiac and respiratory function by drastically decreasing the expression of collagen and significantly reducing levels of phosphorylated Smad3 [83]. Indeed, halofuginone treatment was also shown to enhance myogenesis, a feature that may be relevant to improve muscle regeneration and function in muscular dystrophy [84].

## Other growth factors involved in fibrosis development

### CTGF

The effects of TGFβ on fibrosis can at least be partially mimicked and amplified by CTGF. Increased levels of CTGF have been reported in skeletal muscle from patients with DMD, dystrophic dogs and *mdx *mice [85,86]. One of the most striking properties of CTGF is its ability to induce collagen type 1, α5 integrin and fibronectin in rat kidney fibroblasts, much more potently than TGFβ [87]. Subcutaneous injection of TGFβ can also regulate the production of CTGF in neonatal Swiss mice, while CTGF injection can cause a fibrotic reaction equivalent to that of TGFβ. This fibrotic response is specific to TGFβ and CTGF, and is not mimicked by EGF, PDGF or FGF2 [87]. Although CTGF seems to interact with decorin, which could be regulating its action, the exact role of CTGF in skeletal muscle remains obscure. These results suggest a role for both TGFβ and CTGF in skeletal-muscle remodeling by inducing fibrosis, inhibiting myogenesis and promoting dedifferentiation of myoblasts into myofibroblast-like cells [86].

## Other growth factors involved in fibrosis development

### PDGF family

Another family of growth factors capable of inducing fibroblastic changes in muscle is the PDGF family, which includes PDGF-AA, PDGF-AB, PDGF-BB, PDGF-CC and PDGF-DD. These molecules bind two different PDGF receptors, α and β, and and *in vivo *they can induce neutrophils, macrophages, fibroblasts and smooth-muscle cells to migrate into a wound site and proliferate. *In vitro*, PDGF production can be induced by TGFβ, which also stimulates fibroblasts to contract collagen matrices and differentiate into myofibroblasts [88]. In addition, PDGF can induce c-abl kinase activity, similar to TGFβ, in a non-canonical manner [89]. Thus, it is not surprising that blocking c-abl activity has anti-fibrotic effects. For example, the anti-neoplastic agent imatinib selectively and competitively blocks the ATP binding site of c-abl and several other tyrosine kinases, including PDGF receptors and c-kit, and it diminishes tissue fibrosis in many experimental mouse models of fibrotic disorders [90]. PDGF and its receptors are also upregulated in inflammatory cells and the regenerating fibers of patients with DMD and *mdx *mice. Consequently, administering imatinib to *mdx *mice reduced fibrosis in the diaphragm, and attenuated skeletal-muscle necrosis and inflammation, with concomitant improvements in muscle function [91]. Complementary studies showed that imatinib ameliorated dystrophy in *mdx *mice after exercise, although treated mice also exhibited significant weight loss [92].

### Myostatin

Myostatin, also known as growth differentiation factor (GDF)8, is a TGFβ family member that is specifically expressed in the skeletal-muscle lineage, where its most characterized role is negative regulation of muscle growth. Deficiency of myostatin increases skeletal muscle mass, myofiber diameter and strength [93,94]. Normal myostatin signaling can be counter-regulated by the extracellular protein follistatin [95]. However, myostatin not only regulates the growth of muscle cells, but also regulates muscle fibroblast activation and hence the progression of fibrosis [96]. Indeed, several groups have reported improved regeneration and decreased fibrosis in the absence of myostatin [74]. Similarly, *mdx *mice lacking myostatin displayed less fibrosis in the diaphragm, and were also stronger and more muscular than their normal *mdx *counterparts [97]. As a potential therapeutic approach, immunological neutralization of myostatin in *mdx *mice was shown to attenuate dystrophy by improving muscle regeneration and reducing ECM accumulation [98]. The effects of myostatin may reflect its direct stimulation of proliferation of muscle fibroblasts, or their differentiation into myofibroblasts, induced when myostatin binds to the activin receptor IIB, as seen both *in vitro *and *in vivo*. In fibroblasts, myostatin induces activation of the Smad, p38 MAPK and AKT pathways to promote ECM synthesis [99]. Not surprisingly, injection of recombinant myostatin protein *in vivo *stimulates myofibers to express TGFβ, which can produce the autocrine pro-fibrotic effects described above. Furthermore, TGFβ induces myostatin production, revealing a collaborative action of both pro-fibrotic cytokines on muscle cells. An important consideration is that the physiological role of myostatin in cardiac muscle seems to differ significantly from its role in skeletal muscle. Consequently, myostatin loss does not induce cardiac hypertrophy nor does it modulate cardiac fibrosis in *mdx *mice [81].

Several other factors may also act by modulating myostatin function. For example, the anti-fibrotic, pro-regenerative effects of decorin may derive not only from its ability to neutralize the effects of TGFβ [73], but also from its capacity to antagonize the action of myostatin on both fibroblasts and myoblasts. Decorin is the main proteoglycan present in the ECM of adult muscle. Biglycan is more weakly expressed, although both proteins are augmented in *mdx *muscle [100,101]. Indeed, whereas loss of biglycan in transgenic mice does not affect regenerative capacity, despite inducing delays in new myofiber growth [100], decorin delivery to regenerating muscle via adenoassociated viral (AAV) vectors prevents TGFβ activation and fibrosis [76]. Decorin can also upregulate the expression of the myostatin inhibitor follistatin [74]. Overexpression of insulin-like growth factor (IGF)-1 and the simultaneous loss of myostatin *in vivo *have been found to have interesting synergistic effects on myofiber growth and impaired fibrosis. Local expression of a muscle-restricted IGF-1 transgene accelerates the regenerative process in injured skeletal muscle, modulating the inflammatory response and limiting fibrosis [39]. However, the mechanisms by which these two genes combine to regulate fibrosis and muscle regeneration are still unclear and under study. Overall, myostatin has distinct fibrogenic properties that, when considered in conjunction with other signaling systems, suggests a co-regulatory relationship between TGFβ, CTGF, myostatin and decorin.

### ECM remodeling in muscle repair

Fibrosis can develop as a consequence of dysregulated wound-healing responses and/or excessive deposition of ECM, preventing normal regeneration after tissue injury. However, appropriate ECM deposition is crucial for efficient repair because the ECM surrounding skeletal muscle plays an important role in maintaining the structure and reinforcing the contractile function of muscle. Furthermore, the ECM sequesters and presents heparin-binding growth factors to the fibers, such as hepatocyte growth factor (HGF) and fibroblast growth factor (FGF), and signals to the differentiated fibers through dystroglycan and sarcoglycan complexes [102,103]. Individual components of the ECM have been analyzed in transgenic knockout models after muscle injury or pharmacological intervention, providing important insight into the function of the ECM in normal conditions and during muscle regeneration. In normal muscle, the external lamina is composed of collagen IV, laminin, and heparan sulfate proteoglycans, while the interstitial matrix that surrounds the basal lamina contains collagens I, III and V, fibronectin and perlecan [10,11,102]. The systematic and timely breakdown and replacement of these layers is crucial to ensure complete and rapid repair, while avoiding fibrosis.

### Fibrin(ogen) in muscle repair and fibrosis

A key element of the provisional primary ECM in injured skeletal muscle is fibrinogen and its end-product fibrin, which are here collectively referred to as fibrin(ogen). Excessive and persistent fibrin(ogen) deposition is deleterious to myofiber repair. Transgenic mice that have lost the fibrinolytic proteases uPA and plasmin (see below) exhibit impaired muscle regeneration because of defective fibrinolysis and also have accumulation of fibrin after muscle injury [33,35]. Fibrin(ogen) also accumulates in the muscles of human patients with DMD and in the diaphragm of *mdx *mice (Figure 4A) [27,37]. Importantly, fibrin(ogen) accumulation is also correlated with increasing age and the progression of fibrosis, whereas fibrin(ogen) depletion in *mdx *animals can significantly rescue muscle fibrosis and disease severity [27]. Fibrin(ogen) deposits may serve as important triggers for the inflammatory responses, because of their capacity to bind to the integrin receptor Mac-1 on classically activated M1 macrophages [27] (see above). For example, after the engagement of fibrin(ogen) with Mac-1, macrophages in *mdx *muscle induce the expression of pro-inflammatory chemokines and cytokines that can promote muscle degeneration, including IL-1β, TNFα, IL-6 and MIP-2 [27]. Persistent fibrin(ogen) deposition in dystrophic muscle sustains chronic inflammation, characterized by the increasing presence of alternatively activated macrophages in *mdx *muscle, as dystrophy progresses with age [27]. As described above, M2a macrophages have been shown to promote fibrosis in several different pathogenic conditions [12], thus supporting the notion that fibrin(ogen) may promote fibrosis by sustaining alternatively activated macrophages in dystrophic muscle. Moreover, by binding to the αVβ3 integrin receptor on fibroblasts, fibrin(ogen) can also directly stimulate the expression of collagen [27]. These findings emphasize the concept that fibrin(ogen) is not merely a structural component of the transient matrix formed after injury, but, rather it can further promote tissue inflammation and fibrosis in persistent chronic conditions, as in muscular dystrophy. Finally, the persistence of components of the ECM produced immediately after injury is clearly deleterious to muscle repair, at least in part by promoting fibrosis.

### The importance of matrix metalloproteinases in skeletal-muscle repair

In addition to the direct synthesis of ECM components, efficient muscle repair also requires factors that regulate the proteolytic degradation of the ECM. These molecules include a large family of matrix metalloproteinases (MMPs), calcium-dependent enzymes that specifically degrade collagens and non-collagenous substrates, and their inhibitors, the TIMPs. Proteolysis is also modulated by molecules of the plasminogen activation system [[16,104]. Indeed, MMPs alone or in conjunction with the plasminogen/plasmin system, can degrade ECM components, which are essential for cell migration and tissue remodeling.

The large family of MMPs includes various collagenases (MMP-1, MMP-8 and MMP-13), gelatinases (MMP-2 and MMP-9), stromelysins (MMP-3, MMP-7, MMP-10 and MMP-11), membrane-type metalloproteinases (MMP-14, MMP-15, MMP-16, MMP-17, MMP-24 and MMP-25), and the metalloelastase MMP-12 [16]. MMPs are released from damaged muscle and infiltrating cells in order to disrupt the basement membrane of the fiber, thereby facilitating the recruitment of myogenic, inflammatory, vascular and fibroblastic cells to damaged tissue. However, the function of MMPs is not only controlled by their expression and release from damaged muscle and inflammatory cells. Net MMP activity also reflects the relative amount of activated enzyme. Activation requires proteolytic cleavage of the inactive precursor by either membrane-type (MT)1-MMP [105] or plasmin, and cleavage of their corresponding inhibitors [106]. For example, increased TIMP-1 and TIMP-2 levels mediate a net decrease in protease activity, and thus, matrix accumulation. The serine protease plasmin can directly activate, at least *in vitro*, a group of MMP pro-domains, such as proMMP-1, proMMP-3, proMMP-9, proMMP-10 and proMMP-11, while the activation of proMMP-2 also involves hydrolysis by MT1-MMP during plasmin stimulation. In some cases, active MMPs can also further activate the proMMPs of other MMPs, thereby forming positive feedback loops.

MMPs play an obvious role in the development of fibrosis, because collagen accumulates when its rate of synthesis is greater than the rate of breakdown by MMPs/collagenases; that is, when the balance between TIMP and MMP activity favors TIMPs. In addition to their role in fibrosis, MMPs and serine proteases fulfill many other roles in skeletal-muscle repair and satellite-cell activity. For example, MT1-MMP is presumed to help maintain myofiber integrity, as transgenic mice deficient in MT1-MMP develop centrally nucleated myofibers that are smaller than normal [105]. MMP-13 and MMP-1 also participate in ECM remodeling during muscle repair [16,107,108], while gelatinase MMP-2 affects new fiber formation by promoting collagen IV degradation in the basement membrane during myoblast proliferation, migration and fusion. In addition, NO, which is released by local and invading cells during injury, can act as a pro-inflammatory molecule, and is able to activate MMP-2, which in turn releases HGF. Importantly, blockers of NOS function, such as nitro- L-arginine methyl ester (L-NAME), have been shown to decrease the acute inflammatory reaction and enhance fibrosis *in vivo*, in part by decreasing iNOS, MMP-2 and HGF [109,110].

Another gelatinase, MMP-9, has been attributed a key role in satellite-cell activation during the initial phase of muscle regeneration, in addition to its role in inflammation. Perhaps more importantly, the skeletal muscle of adult *mdx *mice expresses high levels of latent and active MMP-2 and MMP-9, and inhibiting MMP-9 activity was shown to significantly improve regeneration and contractile functions by reducing the structural deterioration of muscle, necrosis, inflammation and fibrosis [16,111-113]. Similar results were achieved in aged *mdx *mice after transplanting modified tendon fibroblasts expressing MMP-9 and placenta growth factor (PlGF). These cells restored a vascular network and diminished collagen deposition, raising the exciting possibility of using similar cell-based or gene therapies to improve muscle function in patients with currently untreatable advanced-stage dystrophies [114]. The protein ADAM12 (a disintegrin and metalloproteinase 12) has been shown to produce benefits in the context of muscle dystrophy and aging by upregulating utrophin and stabilizing the plasma membrane [115]. However, chronically high levels of ADAM12 inhibit satellite-cell responses and delay myoblast differentiation, subsequently leading to skeletal-muscle loss with accelerated fibrosis and adipogenesis in aged *mdx *mice.

### The role of the plasminogen activation system in ECM remodeling during repair of damaged muscle

The activity of MMPs can amplify or synergise in some cases with proteases of the plasminogen activation system in ECM remodeling during tissue repair, particularly due to their capacity to degrade multiple ECM proteins. The plasminogen activation system is centered on an inactive, extracellular serine protease, plasminogen, which is converted into the active enzyme, plasmin, by two plasminogen activators (PAs): tissue-type plasminogen activator (tPA) and urokinase-type plasminogen activator (uPA). Inhibition of the plasminogen activation system occurs at the level of the PAs by PA inhibitor (PAI)-1 or at the level of plasmin, by α2-antiplasmin [104]. The main function of the plasminogen activation system main function is to degrade fibrin [104], but it also fulfills an important role in muscle ECM remodeling by cleaving ECM-associated molecules, liberating and/or activating latent forms of bioactive molecules such as growth factors, angiogenic factors and cytokines (particularly bFGF, TGFβ, VEGF, HGF/SF and certain MMPs). Many of these molecules may also help in the activation of quiescent satellite cells and regulate subsequent myogenic programs.

It is not surprising that components of the plasminogen activation system play important yet distinct roles in muscle regeneration after injury. Whereas it has been shown that both uPA and plasmin activity are necessary for skeletal-muscle regeneration, tPA activity is dispensable, indicating that no redundancy exists in muscle [33,35]. By contrast, the negative role of PAI-1 in muscle regeneration is suggested by the improved muscle repair seen after injury in PAI-1-deficient animals [36]. The plasminogen activation system also plays an important role in muscular dystrophy. For example, increased uPA expression was found in *mdx *muscle, whereas genetic loss of uPA exacerbated dystrophy and impaired muscle function in *mdx *mice [37]. Recent data also suggest that PAI-1 may influence dystrophic muscle in humans and mice, and that the uPA/PAI-1 balance potentially affects muscle fibrosis (our unpublished results). Satellite cells derived from human patients with DMD produce more uPA receptor and PAI-1 and less uPA than do normal satellite cells [116]. BM-derived cells expressing uPA also seem to play a key role in *mdx *muscle repair with transplantation experiments, suggesting that it may act in a number of different ways. Firstly, BM-derived uPA-expressing cells prevent excessive deposition of fibrin through the normal role of uPAs in fibrin degradation. Secondly, BM-derived uPA also promotes myoblast migration, presumably by activating or increasing the availability of growth factors. Finally, it increases infiltration of BM-derived inflammatory cells, especially macrophages into damaged tissue. However, genetic loss of the uPA receptor in *mdx *mice failed to exacerbate muscular dystrophy, suggesting that uPA exerts its proteolytic effects independently of its receptor [37].

Based on all of the emerging data, it is tempting to speculate that the pronounced fibrosis in seen in patients with DMD is related to altered net proteolytic activity in the dystrophic muscles, due to imbalances in expression and activity of PA/MMP system components [101,116]. In turn, this imbalance could provoke the aberrant activation of latent TGFβ, with serious consequences for fibrosis. Although proteolytic processing by plasmin and MMP is important, as are integrins and thrombospondin, the full gamut of mechanisms leading to TGFβ activation is complex and remains poorly understood.

### Other factors affecting muscle ECM remodeling

Stem-cell antigen (Sca)-1 has been shown to influence muscle regeneration by modulating ECM remodeling. Sca-1-deficient mice exhibit defects in muscle regeneration, as Sca-1 is a negative regulator of myoblast proliferation and differentiation *in vitro*. These mice also show enhanced fibrosis after injury, as a result of reduced MMP activity. It is still not clear if Sca-1 acts directly or indirectly to upregulate MMP activity, which could in turn increase matrix breakdown and efficient muscle regeneration, while halting fibrosis [117]. A more recent follow-up study [118] related the increased fibrosis of Sca-1-deficient mice to a reduced recruitment of IgM and C3 to injured muscle, due to a specific reduction in peritoneal IgM-producing B-1a cells. Because Sca-1 also plays a role in the maintenance of progenitor cells [119], it was hypothesized that the reduction in B-1a cells was due to lineage-specific defects in self-renewal. Regardless, recruitment of IgM and C3 after muscle injury is essential for the recognition and clearance of dead cells by phagocytic macrophages and thus, efficient regeneration. Although more detailed studies of the role of IgM and C3 in muscle regeneration and fibrosis are necessary, C3 has already been shown to influence the development of liver fibrosis in other animal models [120].

In aged mice, a Sca-1+, non-myogenic (MyoD-) and non-immunohematopoietic (CD45-) cell population has been proposed to favor fibrosis [121]. These cells are found in aged regenerating muscle, or they can be clonally derived from myoblasts isolated from aged animals or late passage C2C12 cells, although they are uncommon in young animals, in which they seem to retain a greater myogenic potential and express MyoD. Importantly, these cells overexpress fibrosis-associated genes, possibly in a manner regulated by Wnt2. Another recent study identified an increase in a muscle-resident stromal cell (mrSC) population in the muscles of *mdx *mice [122]. Wnt3a was shown to promote the proliferation of mrSCs in culture and the expression of collagen, while exerting the opposite effects on cultured myoblasts. Moreover, injecting Wnt3a into the tibialis anterior muscles of adult wild-type mice significantly increased the mrSC population and collagen deposition. Conversely, injection of the Wnt antagonist DKK1 into the skeletal muscles of *mdx *mice significantly reduced fibrosis. Thus, as described above, the canonical Wnt pathway seems to augment the mrSCs population and to stimulate the production of collagen by these cells. Hence, the Wnt pathway, and potentially mrSCs, are further confirmed as targets for therapies to counteract fibrosis during aging and in various myopathies.

Recent studies have implicated the IGF-family member relaxin in the regulation of MMP expression and fibrosis development. Treatment with relaxin was able to limit fibrosis development after laceration in mice [123]. Mechanistically, relaxin was shown to promote myoblast proliferation and myogenesis *in vitro *by downregulating p21 and upregulating Pax7 expression, while simultaneously increasing expression and activation of several MMPs, an effect abrogated by the MMP blocker GM6001 [123,124]. However, the mechanism of action of relaxin *in vivo *is complicated by its combined effects on myogenesis and fibrogenesis, and its apparent ability to reduce inflammation and promote angiogenesis when injected directly. Similar effects were also seen in aged muscle [124]. Further studies are needed to identify the key target cells of relaxin, the kinetics of its activity, and whether its ability to limit collagen deposition applies to chronic injury and myopathies in addition to acute models of injury.

### Muscle dysfunction and fibrosis in sarcopenia

In addition to the factors already described above, several studies have highlighted additional characteristics of aging skeletal muscle that influence regeneration and fibrosis. For example, a major role for myofibers themselves in controlling the homeostasis of the ECM during age-related sarcopenia has been proposed [125]. Indeed, some of the muscular features associated with aging in humans were reproduced prematurely in a mouse model with the post-natal, muscle-specific deletion of serum response factor (SRF), including type IIB myofiber atrophy, sarcomere disorganization and endomysial fibrosis. Impaired functional and morphological regeneration, in addition to persistent and increased fibrosis, was also seen in this mouse model after cardiotoxin-induced injury. Because in these mice SRF was selectively ablated in post-mitotic myofibers, the impaired regeneration and increased fibrosis was attributed to secondary modifications of the stem-cell niche, coupled with a modified environment that favored the appearance and maintenance of fibrosis. Significantly, SRF protein expression is reduced with aging in mouse and human muscle samples, suggesting a physiological role for this factor in the pathogenesis of sarcopenia.

Studies of the effects of aging on the expression of microRNAs in skeletal muscle of humans found that several members of the Let-7 family were upregulated in older subjects. Because Let-7 family members been related to cell-cycle control, they may function to downregulate other genes involved in muscle-cell proliferation, which in turn might negatively affect the reduced regenerative capacity of muscle seen with aging [126]. Aged human muscle also behaves differently from young muscle after a similar modest level of damage, with dysregulation of a particular set of transcripts including transcripts related to fibrosis and connective-tissue function [127]. The age-specific response to damage seen in older people included higher protein expression for NF-κB, heat shock protein 70 and STAT3 signaling. Increased STAT3-dependent responses in aged versus young muscles have also been reported in humans after resistance exercise, which has being hypothesized to be related to the efficiency of muscle repair and regeneration [128].

## Conclusions

Fibrosis is the end result of a complex series of events that follow tissue damage and inflammation. If this process is faulty, excessive and persistent ECM deposition takes place, and normal tissue is substituted by collagen scar, resulting in tissue dysfunction. Dysregulated muscle repair with persistent fibrosis rather than efficient regeneration plays a prominent role in the clinical decline and reduced life expectancy associated with severe muscular dystrophies, especially in DMD. If the fibrotic tissue could be repaired and the dystrophic muscle be redirected towards regeneration, at least to some extent, thereby preserving muscle integrity, the health of such patients could be considerably improved. Persistent fibrosis represents a major obstacle for successful gene- and cell-based therapies for DMD that aim to restore or replace the dystrophin gene. Thus, modifications of the muscle environment aimed at halting and/or reducing fibrosis in DMD are crucial to attenuate disease progression, and to improve gene delivery and stem-cell grafts in otherwise untreatable patients. Inflammatory cells, particularly macrophages, are crucial in regulating tissue homeostasis and promoting aberrant healing. They are emerging as indispensable players for damage control and tissue remodeling on muscle injury, and as principal mediators of pathological skeletal remodeling in diseases such as the IIMs and the dystrophies. Despite their many functions, skeletal-muscle macrophages remain poorly characterized in molecular terms. Evidence is evolving that muscle macrophages are a heterogeneous population, which probably derive from distinct origins in physiology and pathology. Finally, although there have been many advances in deciphering the variety of pathways involved in normal muscle regeneration and muscular dystrophy-associated fibrosis, these have not yet been translated into effective anti-fibrotic therapies for patients with DMD. It seems likely that single-agent therapies, such as growth-factor administration or antagonism of a single signaling pathway, will have a very weak effect on fibrosis at advanced disease stages in a clinical setting. This is probably due to the redundancy of growth factors and of the cellular participants, ECM components and signaling pathways involved in muscle regeneration/fibrosis, and to the rapid neutralization or elimination of individual agents. Thus, combined strategies will be crucial to combat fibrosis and ameliorate the progression of muscular dystrophy, including systemic delivery of anti-fibrotic agents and gene-corrected cells that could integrate environmental inputs from the host and convert them into biological transmitters.

## Competing interests

The authors declare that they have no competing interests.

## Authors' contributions

All authors contributed to some extent to the writing and editing of the manuscript, and to figure design. All authors read and approved the final manuscript
